# Automatic Detection System for Velopharyngeal Insufficiency Based on Acoustic Signals from Nasal and Oral Channels

**DOI:** 10.3390/diagnostics13162714

**Published:** 2023-08-21

**Authors:** Yu Zhang, Jing Zhang, Wen Li, Heng Yin, Ling He

**Affiliations:** 1 College of Biomedical Engineering, Sichuan University, Chengdu 610065, China; zhangyu5@stu.scu.edu.cn (Y.Z.); jing_zhang@scu.edu.cn (J.Z.); liwen1@stu.scu.edu.cn (W.L.); 2 West China Hospital of Stomatology, Sichuan University, Chengdu 610041, China; phoebeyin@126.com

**Keywords:** velopharyngeal insufficiency, speech disorder, automatic diagnosis, deep learning

## Abstract

Velopharyngeal insufficiency (VPI) is a type of pharyngeal function dysfunction that causes speech impairment and swallowing disorder. Speech therapists play a key role on the diagnosis and treatment of speech disorders. However, there is a worldwide shortage of experienced speech therapists. Artificial intelligence-based computer-aided diagnosing technology could be a solution for this. This paper proposes an automatic system for VPI detection at the subject level. It is a non-invasive and convenient approach for VPI diagnosis. Based on the principle of impaired articulation of VPI patients, nasal- and oral-channel acoustic signals are collected as raw data. The system integrates the symptom discriminant results at the phoneme level. For consonants, relative prominent frequency description and relative frequency distribution features are proposed to discriminate nasal air emission caused by VPI. For hypernasality-sensitive vowels, a cross-attention residual Siamese network (CARS-Net) is proposed to perform automatic VPI/non-VPI classification at the phoneme level. CARS-Net embeds a cross-attention module between the two branches to improve the VPI/non-VPI classification model for vowels. We validate the proposed system on a self-built dataset, and the accuracy reaches 98.52%. This provides possibilities for implementing automatic VPI diagnosis.

## 1. Introduction

Velopharyngeal insufficiency and/or incompetency (VPI) refers to abnormal palatopharyngeal function. The airflow passage between the soft palate and the pharyngeal walls (or adenoids in children) does not close completely [1]. This defect severely affects the patient’s daily life. VPI causes characteristic speech disorders, including hypernasality and nasal air emission [2]. They reduce the clarity of the patient’s speech. It causes situational difficulty and emotional impacts, which affects normal interpersonal communication [3]. Additionally, VPI is closely related to swallowing disorders, which affects daily eating [4]. These symptoms seriously affect the physical and mental health of patients.

According to the causes of the disease, VPI can be classified as congenital VPI or acquired VPI [5]. Congenital developmental malformations, such as cleft palate [6] and congenital myotonic dystrophy [7], can lead to congenital VPI. In terms of acquired VPI, tonsillectomy [8], adenoidectomy [9], palatal sail shortening, or trauma [10] can cause it. VPI treatment requires continuous therapy. Approximately 5–35% of cleft palate patients still suffer from VPI after palatopharyngeal repair surgery [11]. This percentage even reaches 40% if it is determined strictly [12]. Treatment of VPI requires surgery or prolonged voice training. The timely diagnosis of VPI is important for the early treatment of patients and the acquisition of normal speech and language skills for children less than 3 years old [13].

Clinical assessment of palatopharyngeal function can be performed using instruments that allow direct visualization of the palatopharyngeal closure status. These include nasoendoscopy [14], multiview videofluoroscopy [15], and medical imaging methods [16,17,18,19]. Researchers have tried to find some velopharyngeal anatomy or velar shape parameters obtained by these instruments to predict whether the prognostic patients had VPI speech disorders or not [20,21]. The majority of patients with VPI are children. These methods are not child-friendly due to their intrusiveness, the involvement of radiation, and the requirement for patients to be highly cooperative.

The noninvasive approach generally relies on a speech-language therapist’s (SLT) assessment of certain phenomena or intermediate data to diagnose hypernasality or nasal air emission caused by VPI. The mirror-fogging test is used to detect nasal air emission [22]. However, it can be affected by resistance of the nasal airways and only assesses nasal breathing without speech production [23]. Nasalance scores determined by Nasometer are commonly used in clinical practice to supplement assessment of hypernasality [24]. The scores represent the energy ratio of the acoustic signals of the nasal and oral channels. Researchers have noted that nasalance score does not have a fixed evaluation criterion for different languages [23]. The use of the Nasometer for determining nasalance scores exclusively relies on the energy of the acoustic signals, inevitably leading to the omission of specific speech perception information [25].

Clinical assessment using speech perception mostly relies on well-trained SLTs for subjective diagnosis of patient speech [26]. The number of SLTs is low, and training experienced SLTs requires a certain amount of time and money. End-to-end VPI detection algorithms, which employ speech signal processing and deep learning techniques, can effectively provide detection results that assist in a clinical VPI diagnosis. These algorithms eliminate the need for additional complex analysis by SLTs. They are both economical and convenient, effectively addressing the issue of late VPI detection in secondary care facilities or underdeveloped medical areas where speech-language therapists may not be readily available.

The current research on speech-based computer-aided VPI detection algorithms is focused on the automatic detection of speech disorders caused by VPI. In terms of hypernasality speech, the extra nasal resonance is present [27]. The nasal formant has been shown to be present around F1 [28]. The extraction algorithms of nasal formant [29,30] have been studied, and formants related characteristic parameters are utilized in automatic hypernasality detection. They contain group delay function-based acoustic measure (GDAM, the ratio of the absolute value at F1 to that at F2 in group delay spectrum) [31], the cross-correlation value of original speech signals and modified speech signals after pole-defocusing [32], the vowel spectral area (VSA) [33], and spectrum-based features [34,35]. The above methods with nasal formant or formants-related parameters are susceptible to age, gender, and noise. Recently, deep learning methods have been used in the study of automatic hypernasality classification, such as deep RNN [36], CNN [37], and improved BLSTM [38]. To solve the problem of sparse hypernasality speech data, researchers [39,40] attempted to use automatic speech recognition models trained by normal speech for the diagnosis of hypernasality in children. However, the validation datasets do not include data from adult VPI patients. In terms of nasal air emission, there are few automatic detection methods. Nasal air emission and hypernasality due to VPI can occur separately or together [41]. There is also a lack of methods that directly detect VPI automatically.

This paper proposes an automatic VPI detection system that operates at the subject level. The data are the acoustic signals collected from the nasal and oral channels. VPI/non-VPI automatic classification methods at the phoneme level are proposed for consonants and vowels. The results at the phoneme level for the subjects are fused to obtain the VPI/non-VPI detection result at the subject level. The above methods can assist clinicians in VPI diagnosis. Our main contributions are summarized as follows:

(1) An automatic VPI/non-VPI detection system at the subject level based on speech is proposed in this paper. The system takes into account distinctive articulatory symptoms caused by VPI, nasal air emission, and hypernasality. For this purpose, the detection of VPI/non-VPI at the subject level is proposed by integrating the symptomatic manifestations at the phoneme level.
(2) VPI causes a change in the propagation path of the airflow through the vocal tract, which affects the acoustic signals radiated from the nasal and oral cavities. For unvoiced consonants, the power spectral density ratio (PSDR) is calculated to indicate airflow leakage to the nasal cavity relative to the oral cavity. This paper proposes relative prominent frequency description and relative frequency distribution features based on PSDR. They are extracted to characterize the perceived acoustic signals radiated from the nasal cavity relative to those from the oral cavity.
(3) Mathematical models for VPI patients and non-VPI controls on vowel articulation are established in this paper. Based on the discrepancy between VPI patients and non-VPI controls shown in the models, a cross-attention residual Siamese network (CARS-Net) is proposed for VPI/non-VPI classification at the phoneme level for vowels. A cross-attention module is proposed that is embedded in CARS-Net to enhance the ability to extract the discriminating features for VPI and non-VPI classification at the phoneme level for vowels.

## 2. Materials and Methods

### 2.1. Materials

#### 2.1.1. The Collected Phonemes

The specific clinical structural speech disorders caused by VPI are nasal air emission and hypernasality [2]. The vibration source of voiced phoneme production comes from the vocal cord, which is located in front of the palatopharynx in the path of airflow propagation. When patients with VPI produce nonnasalized vowels, some of the airflow carrying the vocal cord wave propagates to the nasal cavity and causes nasal resonance, resulting in hypernasality. In contrast, the production of unvoiced consonants relies on the friction between the airflow and the various articulatory parts of the vocal tract, most of which appear behind the palatopharynx [42]. In this case, the airflow leakage to the nasal cavity does not cause strong nasal resonance. However, compared to voiced phonemes, the unvoiced consonants included in unvoiced phonemes have continuous airflow overflow and reduce the effect of nasal resonance, which results in the nasal air emission symptoms [43].

Nasal air emission symptoms caused by VPI arise from specific types of consonants: plosives, affricates, and fricatives [44]. The airflow exhaled from the lungs during unvoiced consonant production is the source of the nasal air emission symptoms caused by VPI. The plosives and affricates are divided into aspirated and nonaspirated phonemes, depending on the relative size of the airflow delivery. In contrast to the nonaspirated phonemes, the aspirated phonemes produce a distinct airflow during articulation. The fricatives are also pronounced with a distinct airflow output. In this work, the aspirated unvoiced consonants are collected into the dataset (/p/, /t/, /k/, /q/, /c/, /h/, /x/, /sh/, /f/).

Hypernasality symptoms caused by VPI are mainly detected in the articulation of vowels [45]. When nasalized vowels are pronounced normally, the velopharynx is open [46]. To highlight the difference between VPI and non-VPI speech on vowels, nonnasalized vowels are chosen, which have almost complete closure of the palatopharynx during normal articulation. In this work, four nonnasalized vowels are considered (/a/, /e/, /i/, /u/).

#### 2.1.2. Dataset

The data used in this work were collected from volunteers recruited by the Cleft Lip Unit of West China Hospital of Stomatology, Sichuan University. A total of 89 patients with VPI and 46 controls without VPI were included in the dataset. The participants are from 10 provinces in China and aged from 4 to 45. And the average ages (mean ± SD) of the VPI and non VPI groups are 18.64 ± 7.58 and 13.04 ± 7.04, respectively. In terms of sex, there are 66 females and 69 males. Among them, the VPI group consists of 43 females and 46 males, and the non-VPI group consists of 23 females and 23 males. There are 4860 phonemes in the dataset.

The Nasometer II 6450 (kayPENTAX, State of New Jersey, USA) is the acquisition device for the dataset. The two microphones carried by this device are separated by a plate, allowing simultaneous recording of the acoustic signals radiating from the nasal and oral cavities. The nasal channel records the sound of a microphone placed close to the nasal cavity, and the oral channel corresponds to the sound recorded by the microphone placed in front of the oral cavity. The sampling rate is 11,025 Hz.

### 2.2. Overview of the Automatic VPI Detection Method

**Figure 1:**
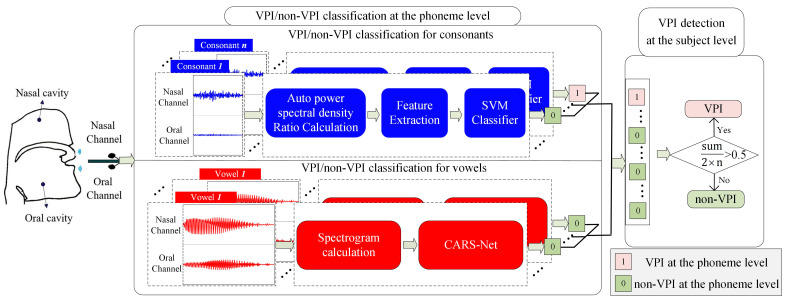
Overview of VPI detection at the subject level.

An automatic VPI/non-VPI detection system at the subject level is proposed in this work. As shown in Figure 1, the process is divided into two steps.

Step 1: VPI/non-VPI classification at the phoneme level.

Due to the different effects of VPI on the pronunciation of unvoiced consonants and vowels, different classification methods are proposed for consonants and vowels, as described below.

(1) VPI/non-VPI classification at the phoneme level for consonants: The relative prominent frequency description and relative frequency distribution are extracted based on the power spectral density ratio sequence. They are combined with a support vector machine classifier to implement the VPI/non-VPI consonant classification model.
(2) VPI/non-VPI classification at the phoneme level for vowels: A cross-attention residual Siamese network (CARS-Net) is proposed in this paper to achieve end-to-end VPI/non-VPI speech classification at the phoneme level for vowels.

Step 2: Automatic VPI detection at the subject level by the voting method.

The VPI/non-VPI classification results for all phonemes of each subject are aggregated through voting to obtain the VPI detection results at the subject level. A subject is classified as a VPI patient if more than half of the participating phonemes are classified as VPI speech.

### 2.3. VPI/Non-VPI Classification Methods at the Phoneme Level for Consonants

**Figure 2:**
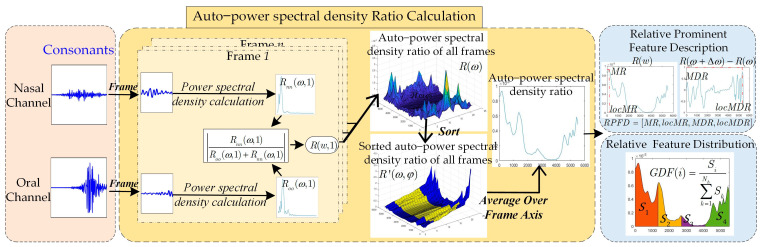
Feature extraction process for consonants.

The proposed automatic VPI/non-VPI speech classification method for consonants is shown in Figure 2. The power spectral density of the acoustic signals radiated from the nasal cavity and oral cavity is approximated by that of the acoustic signals collected by the equipment. Relative prominent frequency description (RPFD) features and relative frequency distribution (RFD) features between the acoustic signals of nasal and oral channels are obtained. They are extracted to model the relative sounds produced by airflow leakage to the nasal cavity. SVM classifier is used to discriminate unvoiced consonant production with and without symptoms caused by VPI.

#### 2.3.1. Power Spectral Density of Radiated Signals from the Nasal Cavity and Oral Cavity

The power spectral density ratio of the acoustic signals of the nasal channel and oral channel is calculated to discriminate unvoiced consonants with and without nasal air emission symptoms caused by VPI. The acoustic signals recorded by the microphones do not only contain the acoustic signal radiated from one cavity, even if there is a separator plate blocking the acoustic signal transmission. This subsection describes the mathematical derivation of the power spectral density of the collected acoustic signals of one channel to approximate the power spectral density of the radiated acoustic signals from the corresponding cavity.

The consonant segments of two-channel acoustic signals collected by microphones in front of the nasal and oral cavities are divided into frames with a frame length of 20 ms and a frame shift of 6 ms. The *i*-th frame signals of the oral channel and the nasal channel are denoted as $x_{o_{i}}\left(t\right)$ and $x_{n_{i}}\left(t\right)$.

Assuming the signal attenuation of the microphone with respect to the other channel is a linear attenuation; the relationship between the collected signals and the radiated signals is as shown in the following equations.

(1) $$\{\begin{array}{c} x_{o_{i}}\left(t\right)=o_{i}\left(t\right)+\alpha \cdot n_{i}\left(t\right)\\ x_{n_{i}}\left(t\right)=\beta \cdot o_{i}\left(t\right)+n_{i}\left(t\right) \end{array}$$

where $o_{i}\left(t\right)$ and $n_{i}\left(t\right)$ represent the signals radiated from the oral and nasal cavities, respectively. β and α are the attenuation coefficients of the baffle for oral and nasal channel acoustic signals, respectively, and β can be regarded as approximately the same as α.

The pronunciation of the unvoiced consonants is based on the friction between the airflow and the vocal organs. When patients with VPI produce unvoiced consonants, the friction factor generated by the airflow overflowing the nasal cavity is different from that of the oral cavity. Therefore, the frictional sounds radiated from each of the two cavities are regarded as uncorrelated signals in this paper. Then, the autocorrelation functions of $x_{o_{i}}\left(t\right)$ can be derived as follows.

(2) $$\begin{array}{l} r_{oo}\left(m,i\right)=\sum^{\;}x_{o_{i}}\left(t\right)x_{o_{i}}\left(t+m\right)\\ =\sum^{\;}\{o_{i}\left(t\right)o_{i}\left(t+m\right)+\alpha o_{i}\left(t\right)n_{i}\left(t+m\right)+\alpha n_{i}\left(t\right)o_{i}\left(t+m\right)+\alpha^{2}n_{i}\left(t\right)n_{i}\left(t+m\right)\}\\ =\sum^{\;}o_{i}\left(t\right)o_{i}\left(t+m\right)+\sum^{\;}\alpha^{2}n_{i}\left(t\right)n_{i}\left(t+m\right)\\ =s_{oo}\left(m,i\right)+\alpha^{2}s_{nn}\left(m,i\right) \end{array}$$

Similarly, the autocorrelation functions of $x_{n_{i}}\left(t\right)$ can be obtained as,

(3) $$r_{nn}\left(m,i\right)=s_{nn}\left(m,i\right)+\alpha^{2}s_{oo}\left(m,i\right)$$

where $r_{oo}\left(m,i\right)$ and $r_{nn}\left(m,i\right)$ represent the autocorrelation functions of $x_{o_{i}}\left(t\right)\;$ and $x_{n_{i}}\left(t\right)$, respectively. And $s_{oo}\left(m,i\right)$ and $s_{nn}\left(m,i\right)$ represent the autocorrelation functions of $o_{i}\left(t\right)\;$ and $n_{i}\left(t\right)$, respectively.

Since the value of $\alpha^{2}$ tends toward zero more than α, we can assume $\alpha^{2}$ equals zero. According to well-known Wiener–Khinchin theorem, the power spectral density of the radiated signals from the nasal and oral cavities can be obtained by applying the Fourier transform to Equations (2) and (3), as shown in the following equations.

(4) $$\{\begin{array}{c} R_{nn}\left(\omega ,i\right)=FFT\left(s_{nn}\left(m,i\right)+\alpha^{2}s_{oo}\left(m,i\right)\right)\approx \;FFT\left(s_{nn}\left(m,i\right)\right)=S_{nn}\left(\omega ,i\right)\\ R_{oo}\left(\omega ,i\right)=FFT\left(s_{oo}\left(m,i\right)+\alpha^{2}s_{nn}\left(m,i\right)\right)\approx \;FFT\left(s_{oo}\left(m,i\right)\right)=S_{oo}\left(\omega ,i\right) \end{array}$$

where $R_{oo}$ and $R_{nn}$ represent the power spectral densities of $x_{o_{i}}\left(t\right)\;$ and $x_{n_{i}}\left(t\right)$, respectively. And $S_{oo}$ and $S_{nn}$ represent the power spectral densities of $o_{i}\left(t\right)\;$ and $n_{i}\left(t\right)$, respectively.

Then, the modulus of the approximate power spectral density ratio of the radiated signals from the nasal and oral cavities of the *i*-th frame  R(ω,i) can be obtained, as shown in the following equation.

(5) $$R\left(\omega ,i\right)=\left|\frac{S_{nn}\left(\omega ,i\right)}{S_{oo}\left(\omega ,i\right)+S_{nn}\left(\omega ,i\right)}\right|=\left|\frac{R_{nn}\left(\omega ,i\right)}{R_{oo}\left(\omega ,i\right)+R_{nn}\left(\omega ,i\right)}\right|$$

where |·| represents the modulo operation. 

#### 2.3.2. Calculation of the Average Power Spectral Density Ratio of the Acoustic Signals Radiated from Nasal and Oral Cavities

To remove outliers caused by random noise, the ratios R(ω,φ) of specifical frequencies ω ordered on the frame axis from smallest to largest and are denoted as $R^{\prime }\left(\omega ,\gamma \right)$, where γ is the ranking position of the specified frequency. The average of the values located between the upper quartiles and lower quartiles is taken as the average power spectral density ratio, as shown in (6).

(6) $$R\left(\omega \right)=\frac{\sum_{k=[\frac{N_{f}}{4}]}^{\left[\frac{N_{f}*3}{4}\right]}R^{\prime }\left(\omega ,k\right)}{[\frac{N_{f}*3}{4}]-[\frac{N_{f}}{4}]+1}$$

where $N_{f}$ represents the number of frames and [·] represents rounding up operation.

#### 2.3.3. VPI Consonant Production Feature Extraction

(1) Relative prominent frequency description between the acoustic signals of nasal and oral channels

The generation of aspirated consonants is directly caused by the friction between the airflow and the gap of the cavity. The intensity of the sounds is positively correlated with the gas dynamic pressure. When patients with VPI produce unvoiced consonants, the airflow overflows into the nasal cavity. Assuming a constant cavity gap and lung dynamics, the relative gas dynamic pressure in the nasal cavity is increased, while the oral gas dynamic pressure is relatively weakened.

In this paper, we extract the maximums of the spectral density ratio (MR) and its first-order differential  (MDR) to characterize the relative air flow to produce acoustic signals, as shown in (7)–(9).

(7) MR=max(R(ω))

(8) MDR=max(R(ω+Δω)−R(ω))

(9) $$\Delta \omega =\frac{f_{s}}{N_{s}}$$

where $f_{s}$ stands for the sampling rate in the time domain, and $N_{s}$ is the number of frequency sampling points. In this paper,$\;N_{s}$ is set to 256.

In addition, the sound quality is related to the shapes of gaps and cavities, which are different in the nasal and oral channels. Therefore, the two cavities produce different frictional noises. The locations of MR and MDR are extracted to reflect the differences in the frequency domain.

(10) $$locMR=arg\underset{\omega }{\max}\left(R\left(\omega \right)\right)$$

(11) $$locMDR=arg\underset{\omega }{\max}\left(R\left(\omega +\Delta \omega \right)-R\left(\omega \right)\right)$$

The above three features are concatenated to form a relative prominent frequency description (RPFD), as shown in the following formula.

(12) RPFD=[MR,locMR,MDR,locMDR]

(2) Relative frequency distribution between the acoustic signals of nasal and oral channels

Aspirated consonants are produced by turbulence, which is generated by friction between the airflow and the cavity gap. The different gap shapes of the nasal and oral cavities create different tuning effects. As a result, the frequency band distributions of the signals radiated from the two cavities are different. When patients with VPI produce aspirated consonants, compared with patients without VPI, the spectral density ratio is enhanced compared to the value at the frequency of the signal radiated from the nasal cavity. This is reflected by the difference in the distributions of the power spectral density ratio. 

The frequency band of the power spectral density is linearly divided into $N_{h}$ subbands. The percentage of each subband area to the total area of the frequency band of the power spectral density ratio is calculated to reflect the relative frequency distribution (RFD), as shown in (13).

(13) $$RFD\left(i\right)=\frac{\sum_{\omega =\frac{\left(i-1\right)*fs}{N_{h}*2}}^{\frac{i*fs}{N_{h}*2}}R\left(\omega \right)}{\sum_{\omega =0}^{fs/2}R\left(\omega \right)}$$

where $N_{h}$ is the total number of frequency sub-bands. In this work, $N_{h}$ is set to 4.

### 2.4. VPI/Non-VPI Classification Methods at the Phoneme Level for Vowels

#### 2.4.1. CARS-Net Proposed for VPI/Non-VPI Vowels Classification 

**Figure 3:**
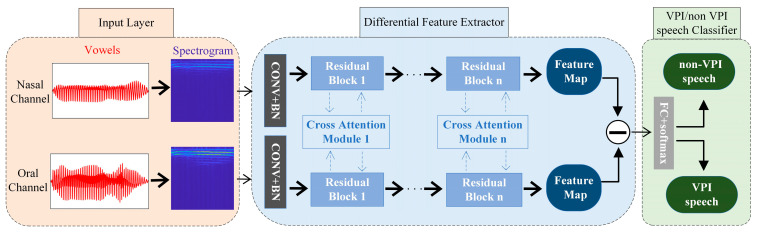
Cross-attention residual Siamese network for VPI/non-VPI classification for vowels.

In this paper, a cross-attention residual Siamese network (CARS-Net) is proposed to achieve automatic VPI/non-VPI classification for vowels. Based on the articulation principle of VPI speech, two-channel acoustic signals from oral and nasal cavities are collected for automatic classification. Compared with a single-input network, the Siamese network structure has dual inputs and is suitable for extracting differences in acoustic signals from the oral and nasal cavities. The network structure proposed in this paper is shown in Figure 3.

As shown, CARS-Net contains three parts: the input layer, difference feature extractor (DFE), and VPI/non-VPI speech classifier. First, the acoustic signals of vowels are collected by two microphones in front of the oral cavity and nasal cavity. They are transformed into spectrograms as the network inputs. Then, the inputs go through the DFE to produce the difference feature map. The DFE contains two branching networks for the two inputs. The two branching networks are linked by the cross-attention module proposed in this paper. Finally, the difference feature map is fed into the VPI/non-VPI speech classifier for automatic classification. The next four sections describe the details of the three parts and loss function for training the network.

#### 2.4.2. Input Layer

The oral and nasal channels of vowels are transformed into spectrograms by framing and short-term Fourier transform operations, and they are then used as inputs to the network. 

**Figure 4:**
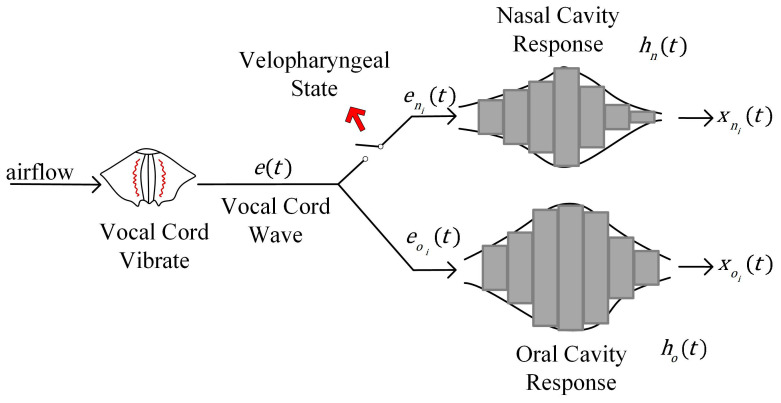
Schematic diagram of the vowel pronunciation with VPI or not.

A schematic diagram of the vowel production process is shown in Figure 4. e(t) represents the vocal cord wave signal generated at the vocal cords. The airflow carrying the vocal cord waves diverges at the pharyngeal wall, partly to the oral cavity and partly to the nasal cavity, denoted as $e_{n_{i}}\left(t\right)$ and $e_{o_{i}}\left(t\right)$, respectively. $h_{n_{i}}\left(t\right)$ and $h_{o_{i}}\left(t\right)$ represent the system response generated by nasal and oral cavity, respectively.

There is a discrepancy between the two channel signals for VPI patients and controls. When a person without velopharyngeal insufficiency (VPI) vocalizes, the switch shown in the figure is open, indicating that the palatopharynx is closed. However, in the case of a VPI patient vocalizing, the switch is closed, indicating that the palatopharynx is not fully closed, thus allowing air to flow into the nasal passage. The system responses include the resonant and radiative effects of the resonant cavity on the vocal cord waves, which can be reflected in the spectrograms of vowels. Furthermore, the spectrograms of vowels change over time compared to the spectrum. In this work, the spectrograms used as the inputs to the network may allow the network to extract the distinguishable features between VPI and non-VPI vowels.

#### 2.4.3. Difference Feature Extractor

Difference Feature Extractor (DFE) is the backbone network of CARS-Net. DFE is mainly used to extract deep features of the input image for subsequent classification. The DFE uses a Siamese-like network structure with two branching networks to extract the differences in the resonance response features contained in spectrograms of the two acoustic signals of the nasal channel and the oral channel.

The two branching networks of the DFE extract the deep features of the two input spectrograms of the nasal and oral cavities. The two branching networks in CARS-Net use a ResNet-18 architecture consisting of residual blocks. The residual block [47] is proposed to alleviate the problem of gradient disappearance due to increasing depth in deep convolutional neural networks by using jump connections.

**Figure 5:**
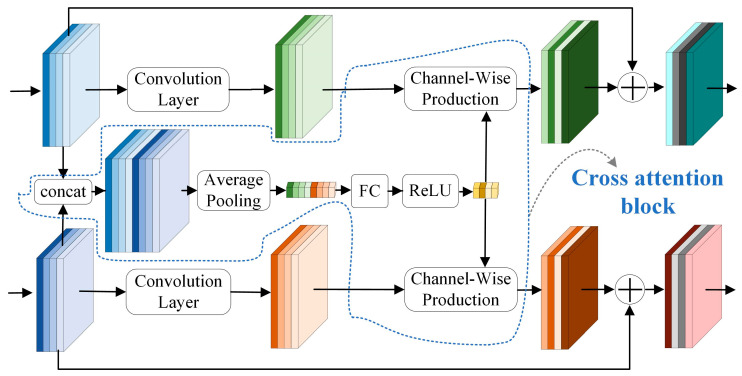
Cross-attention module.

In this paper, a cross-attention module (CA module) is designed to link the two branching networks. This means embedding a cross-attention block in the residual blocks in the two branching networks. The two input acoustic signals are correlated, and they can be expressed as two signals after obtaining different system responses for a homologous signal, as shown in (17). The traditional Siamese network structure has no connection between the two branching networks. The two branching networks share weights but process the two inputs independently without utilizing the correlation information between the two inputs. The CA module is implemented by coupling the intermediate feature maps of the two channels. This allows the originally independent branching networks to be linked in the feature extraction process, enhancing the model’s ability to control the global information. Figure 5 shows the detailed structure of the proposed cross-attention block embedded in the residual block.

The input feature maps of the *i*-th residual block in each of the two backbone networks are denoted as $F_{n\_i}\in R^{H_{i}\times W_{i}\times C_{i}}\;\mathrm{and}\;F_{o\_i}\in R^{H_{i}\times W_{i}\times C_{i}}$ respectively. The two input feature maps are concatenated in the channel dimension to obtain the fused feature maps, $F_{cct\_i}\in R^{H_{i}\times W_{i}\times 2C_{i}}$. Average pooling is performed on $F_{cct\_i}$ in the channel dimension. Pooling operations are used to refine the global information and reduce the number of operations [48]. 

Afterward, a fully connected layer operation is performed to output a weight vector of dimension $C_{i}^{\prime }$. The nonlinearity of the fully connected layer can better fit the information of each channel of the feature maps. Then, a ReLU activation function is used to obtain the final weight vector $W_{i}\in R^{1\times 1\times C_{i+1}}$.

The output feature maps of the feature extraction module $(F_{n\_i}^{\prime }\in R^{H_{i+1}\times W_{i+1}\times C_{i+1}}$, $F_{o\_i}^{\prime }\in R^{H_{i+1}\times W_{i+1}\times C_{i+1}})$ are numerically dot-multiplied with the weight vectors in the channel dimension to obtain the weighted feature maps ($M_{n\_i}\in R^{H_{i+1}\times W_{i+1}\times C_{i+1}},\;M_{o\_i}\in R^{H_{i+1}\times W_{i+1}\times C_{i+1}})$.

Finally, the weighted feature maps are used to replace the original feature maps with the input feature maps in order to obtain the output feature maps of the *i*-th block, as shown in (14).

(14) $$\{\begin{array}{c} F_{n\_i+1}=F_{n\_i}+M_{n\_i}\\ F_{o\_i+1}=F_{o\_i}+M_{o\_i} \end{array}$$

where $F_{n\_i+1}$ is both the output of the *i*-th residual block and the input of the *i* + 1-th residual block.

#### 2.4.4. VPI/Non-VPI Classifier at the Phoneme Level for Vowels

CARS-Net uses a fully connected layer for end-to-end VPI/non-VPI classification instead of threshold judgment, in contrast to the traditional Siamese network. The difference feature map obtained by the two branching networks is flattened into vectors, which are then fed sequentially into a fully connected layer and a softmax layer to achieve binary classification. The process is shown in (15).

(15) $$output=softmax(FC(flatten(F_{n\_final}-F_{o\_final})$$

In the traditional Siamese network structure, the Euclidean distances of the final feature maps obtained from the two backbone networks are calculated for threshold classification, as shown in (16).

(16) $$d=||F_{n\_final}-F_{o\_final}||{}_{2}$$

where d represents the Euclidean distance of the final feature maps, and $||\cdot ||{}_{2}$ is the L2 norm.

This classification method compresses the feature differences between the two channel signals into a single value, with an eye on the overall differences between the dual-channel inputs. This is equivalent to assigning the same weight to each local feature difference in the classification. In contrast, in the classification task of this work, the individual detailed differences in the two input spectrograms reflect different system response features. Different pronunciation system response features should also make different contributions to the classification. The fully connected layer is a nonlinear operation that assigns different classification weights to features at different positions in the difference feature map.

#### 2.4.5. Loss Function

The loss function of CARS-Net consists of two parts, contrast entropy loss and cross-entropy loss, as shown in (17).

(17) Loss=loss1+loss2 

where loss1 is the cross-entropy loss for the VPI/non VPI classification results, and loss1 is the contrast entropy loss.

The cross-entropy loss captures the final classification accuracy, as shown in (18).

(18) $$loss1=-\left(y_{i}\log\left(P\left({\hat{y}}_{i}\right)\right)+\left(1-y_{i}\right)\log\left(\left(1-P\left({\hat{y}}_{i}\right)\right)\right)\right)$$

where $P\left({\hat{y}}_{i}\right)$ is the predicted probability of existing VPI for the *i*-th sample. 

Scholars [49] have designed the contrast entropy function as a loss function for network training in a traditional Siamese network based on the differences in the feature maps of the two branching networks. The contrast entropy loss provides direction for the overall feature extraction, as shown in (19).

(19) $$loss2=-\left(y_{i}d^{2}+\left(1-y_{i}\right)\max{\left(\mathrm{margin}-d,0\right)}^{2}\right)$$

where the margin takes 2, and $\;y_{i}$ is the label of the *i*-th sample.

## 3. Results and Analysis

### 3.1. Experiment Settings

For VPI/non-VPI classification at the phoneme level for consonants, two feature sets, RPFD and RFD, are proposed based on a power spectral density ratio sequence. The VPI/non-VPI classification model at the phoneme level for unvoiced consonants is then obtained by the SVM classifier. 

For VPI/non-VPI classification at the phoneme level for vowels, a network structure, CARS-Net, is proposed. The parameters are initialized using the He initialization method [50] for training CARS-Net. The optimizer is selected as Adam [51]. Regarding the hyperparameter settings, the number of epochs is set to 100, the batch size is 64, and the learning rate is 0.001. The size of the spectrogram is 64 × 64.

The VPI/non-VPI classification experiment at the phoneme level for consonants and vowels uses 10-fold cross-validation. To decrease the error introduced by the detection results of individual consonants or vowels, a voting mechanism is used for the classification of the VPI for each patient/subject. The classification results of all phonemes participating in the experiment for a given subject are counted. A subject is classified as a VPI patient if more than half of the phonemes of the subject were identified as VPI speech.

### 3.2. VPI Detection Results at the Subject Level

Table 1 shows the VPI/non-VPI classification results at the subject level, represented by six classification evaluation metrics, namely, accuracy, precision, recall, F1-score, true negative rate (TNR), and false positive rate (FPR). Accuracy represents the ratio of the number of all correctly predicted VPI and non-VPI samples to the total number of samples in the dataset. Precision represents the proportion of all subjects predicted to be VPI patients who are actually VPI patients. Recall represents the proportion of VPI patients in the dataset who are correctly classified. The F1-score is the summed average of precision and recall, which combines the values of precision and recall and ranges from 0 to 1. The closer the value is to 1, the better the performance of the classification model.

As shown in Table 1, the accuracy of VPI detection at the subject level reached 98.52% based on the method proposed in this paper. These are the results for the binary classification of all VPI and non-VPI subjects in the dataset. The precision and recall were 97.8% and 100%, respectively. The F1-score reached 98.89%. It is shown that the proposed method can correctly detect all VPI patients in the dataset. In clinical diagnosis, misdiagnosis and omission can affect the treatment of VPI patients, leading to serious medical errors. A method with 0% missed detection and 2.2% misdiagnosis on this dataset might play a supporting role in clinical VPI diagnosis.

As shown in Table 1, the TNR and FPR are 95.65% and 4.35%, respectively. This means that 95.65% of the subjects in the dataset that are non-VPI controls were correctly predicted. In the clinic, false-positive diagnoses can lead to misdiagnosis and delay the treatment that the patient should receive. A low false positive rate is important in the clinical diagnosis of VPI.

In terms of the VPI/non-VPI classification results at the subject level, the voting method can improve the robustness of the automatic VPI/non-VPI detection system compared to using individual phoneme classification results. In the clinical diagnosis of VPI, speech therapists synthesize the auditory perception of a whole paragraph rather than a single phoneme. This paper uses a voting mechanism to perform automatic subject level VPI/non-VPI detection based on our proposed phoneme level detection algorithm. This method is consistent with clinical diagnosis.

### 3.3. VPI/Non-VPI Classification Results at the Phoneme Level for Consonants

This subsection discusses the VPI speech classification results for consonants. In the VPI/non-VPI classification method for consonants, two sets of features, RFD and RPFD, are extracted based on the power spectral density ratio sequence of the two-channel acoustic signals collected from the nasal and oral cavities, respectively. Table 2 shows the average VPI speech classification accuracy at the phoneme level for consonants based on RPFD, RFD, and combinations of the two sets of features with three different classifiers.

As shown in Table 2, the VPI speech classification accuracies of consonants based on RPFD range from 76.19% to 78.82%. The classification accuracies of VPI speech based on RFD features range from 80.10% to 84.81%. RFD has more differentiation of classification than RPFD for VPI speech and non-VPI speech) in this dataset. 

Both RPFD and RFD are features extracted on power spectral density ratio sequences of the nasal and oral channel acoustic signals. RPFD is extracted to reflect the most prominent and abrupt frequencies of the acoustic signals of the nasal channel compared to the acoustic signals of the oral channel, while RFD is a reflection of the relative frequency distribution of the two channels. RPFD is a reflection of specific frequency values, which are more affected by noise and less robust than RFD. As shown in the VPI speech classification results, the accuracy of the classification model based on RFD features is higher than that of the classification model based on RPFD features.

As shown in Table 2, the VPI speech classification accuracy of the combination of RPFD and RFD is 83.30–85.00%, which is higher than that of single-set features. The two sets of features, RPFD and RFD, complement each other for VPI and non-VPI speech binary classification model descriptions.

### 3.4. VPI/Non-VPI Classification Results at the Phoneme Level for Different Consonants

This subsection discusses the results of VPI speech classification for different consonants to investigate the effect of articulatory processes and articulatory organs on the proposed VPI consonant classification method.

**Figure 6:**
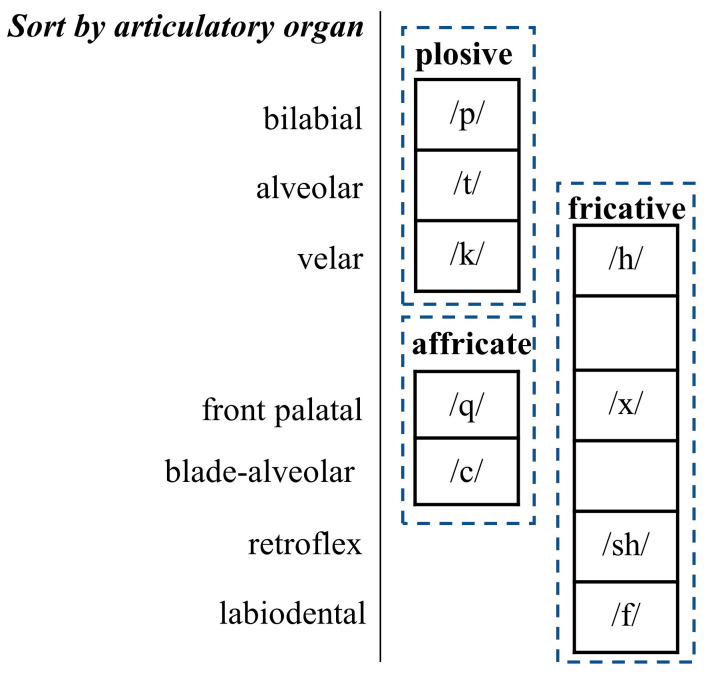
Classification of the unvoiced consonants by the articulation organs.

Three types of consonants with different articulatory processes are involved in this experiment: the aspirated plosives (/p/, /t/, /k/), aspirated affricates (/q/, /c/), and fricatives (/h/, /x/, /sh/, /f/). These consonants are classified by articulatory organ into bilabial (/p/), alveolar (/t/), velar (/k/, /h/), front palatal (/q/, /x/), blade alveola (/c/), retroflexes (/sh/), and labiodental (/f/) as shown in Figure 6.

Table 3 shows the VPI speech classification results for different consonants using the method proposed in this paper.

As shown in Table 3, the VPI speech classification accuracy for plosives (/p/, /t/, /k/) ranges from 73.13% to 81.54%; the VPI consonant classification accuracy for affricates (/q/, /c/) ranges from 86.58% to 91.00%; and the accuracy for fricatives (/h/, /x/, /sh/, /f/) ranges from 81.05% to 92.12%. The accuracy of VPI consonant classification for plosives is lower compared to that for affricates and fricatives.

VPI leads to a partial overflow of air to the nasal cavity and a decrease in oral airflow. After the deblocking process of affricates and fricatives, the airflow passes through small gaps shaped by the articulatory organs [52], and the plosives involve a complete closure and lack the air friction process [52].Therefore, the reduction of oral airflow has less effect on the pronunciation of plosives than affricates and fricatives.

As shown in Table 3, for the same articulatory organ (/q/, /x/), the fricative-based VPI speech classification accuracy (/x/) is 92.12%, which is higher than the affricate-based VPI speech classification accuracy (/q/) of 91.00%. In terms of vocalization duration, that of fricatives is the longest, that of affricates is the next shortest, and that of plosives is the shortest [52,53]. As the duration of the vocalization increases, the airflow continues to spill into the nasal cavity due to VPI. This results in a more pronounced decrease in air pressure in the oral cavity. At this time, the change in the relative spectrum distribution of radiated signals from the nasal and oral cavities is greater. Therefore, the non-VPI and VPI speech signals are more distinguishable according to the fricative-based features proposed in this paper.

As shown by the VPI speech classification results from the perspective of different articulatory organs in Table 3, the front palatal-based (/q/, /x/) classification accuracy is optimal with a maximum of over 91%. The highest blade alveolar-based, retroflex-based, and labiodental-based classification accuracies are above 85%. The velar (/h/) accuracy obtained the lowest accuracy, only 81.05%, among all the affricates and fricatives.

The front palatals (/q/, /x/) are pronounced with the front of the tongue against or near the front of the hard palate, where the airflow is obstructed and then formed. The location of articulatory deblocking for the front palatals (/q/, /x/) is at the hard palate. VPI causes a gap in the plane where the palatopharynx and hard palate are supposed to form, causing it to further interfere with the deblocked articulation process of the hard palate. This makes front palatals (/q/, /x/) more distinguishable in VPI/non-VPI classification at the phoneme level for consonants.

The soft palate is the deblocking site for the velar (/h/). The soft palate is located closer to the palatopharynx than the rest of the affricates and fricatives. VPI results in less impact on the reliance on soft palate position to deblock vocalization when airflow is shunted in the palatopharynx. This results in lower differentiation between the non-VPI and VPI pronunciations of the velar (/h/).

### 3.5. Effect of the Parameter of RFD on VPI/Non-VPI Classification for Unvoiced Consonants

$N_{h}$ is the number of subbands that are linearly divided from the whole frequency band. The different values of $N_{h}$ divide the frequency band into different numbers of frequency bands in the RFD. This subsection explores the effect of different values of $N_{h}$ on the VPI/non-VPI classification for consonants. 

As shown in Table 4, the VPI/non-VPI speech classification accuracies are 84.81%, 84.32%, and 84.80% when $N_{h}$ is taken as 4, 8, and 16, respectively. The increase in $N_{h}$ does not have an improvement on the accuracy of VPI/non-VPI speech classification at the phoneme level for consonants. 

The consonant pronunciation of VPI patients presents stronger nasal radiation acoustic signals than that of non-VPI controls. It produces a change in the percentage of the concentrated frequency band of the oral radiation acoustic signals in the whole frequency band. The frequency of most unvoiced consonants in Mandarin radiated from the oral cavity are higher than 4000 Hz [54]. When $N_{h}$ is taken as 4, a frequency band has a frequency range of 4134–5502.5 Hz, which is almost coincident with the concentrated frequency bands of the consonants radiated from the oral cavity. Therefore, $N_{h}$ takes an empirical value of 4 in this paper.

### 3.6. Analysis of VPI Speech Classification Results Based on Different Vowels 

When nonnasalized vowels are produced by VPI patients, the vocal cord wave spills into the nasal cavity, which does not occur without VPI. In this paper, CARS-Net is proposed to perform automatic VPI/non-VPI speech classification for vowels. It captures the different feature maps of the spectrum of the acoustic signals of the nasal and oral channels to distinguish vowels produced by patients with VPI and without VPI. Table 5 shows the results of automatic VPI/non-VPI speech classification for different vowels.

From the VPI/non-VPI speech detection results of the four vowels (/a/, /i/, /e/, and /u/) shown in Table 5, the classification accuracies of both /i/ and /u/ exceed 93%, while the classification accuracies of /a/ and /e/ are approximately 90%. The recall of 92.92% and 93.55% for /a/ and /e/ is also lower than the average recall of over 95% for /i/ and /u/. This indicates that the model is more sensitive in classifying the VPI/non-VPI speech of /i/ and /u/ than /a/ and /e/. This is consistent with the conclusions reached in the works [36,37], which does not provide an explanation for this phenomenon. In this paper, it is proposed that this phenomenon is related to the level of tongue position when pronouncing vowels.

The shapes of the tongue and lip constitute different oral resonator shapes, thus producing different vowel sounds. The position of the tongue describes the vertical distance between the upper surface of the tongue and the palate [55]. According to the position of the tongue, /a/ is a low vowel, /e/ is a semihigh vowel, and /i/ and /u/ are high vowels. The higher the tongue position, the closer the tongue is to the palate, and the narrower the airflow passage between the tongue and the palate. A narrow passage impedes the passage of airflow more than a wide passage.

If velopharyngeal function is normal, the airflow only moves toward the oral cavity when nonnasalized sounds are produced. In the presence of VPI, the airflow is directed to both the nasal and oral cavities. When producing higher lingual vowels, the narrower airflow passage between the tongue and the palate may force more airflow toward the nasal cavity. This results in more vocal cord waves flowing into the nasal cavity and fewer into the oral cavity. The difference between the acoustic signals of the nasal and oral channels is more distinguishable between VPI high vowels and non-VPI high vowels. This may explain why the CARS-Net proposed in this paper is more sensitive to high vowels (/i/, /u/) in automatic VPI/non-VPI speech classification for vowel tasks

### 3.7. Effectiveness of the Cross-Attention Module

To verify the effectiveness of the proposed cross-attention module in this paper, ablation experiments are conducted in this section. Table 6 shows the comparison of the model prediction results after training the network without and with the CA module using the same hyperparameters.

As shown in Table 6, for each different vowel, the model prediction results of the network model containing the CA-module outperformed the training model without the CA-module in all four metrics (accuracy, precision, recall, and F1-score), showing the effectiveness of the proposed CA-module for VPI recognition in this paper. This shows that the CA-module can improve the ability of the traditional Siamese network to extract the correlation features of the two channel acoustic signals for VPI/non-VPI speech classification.

The accuracy of the network model with the CA module improved by 9.49%, 2.93%, 4.46%, and 1.04% on /a/, /e/, /i/, and /u/, respectively, compared with the network model without the CA module. On the one hand, the CA module improved the VPI/non-VPI speech classification results for /a/ most significantly, which is the vowel with the lowest sensitivity in VPI/non-VPI speech classification among the four vowels involved in the experiment. On the other hand, although the network models containing the CA module show different improvements in VPI/non-VPI speech classification based on all four vowels, the VPI speech recognition accuracies of /a/ and /e/ are still lower than those of /i/ and /u/ when using the CA module. The CA module improves the overall recognition effectiveness of the network for VPI speech without changing the relative effectiveness for different vowels. This further indicates that the VPI/non-VPI speech classification method for vowels proposed in this paper has higher discriminative power for /i/ and /u/ than for /a/ and /e/.

### 3.8. Validation of the Loss Function

The loss function used in this paper contains two parts, the cross-entropy loss (loss1), for binary classification results, and the contrast entropy loss (loss2) between the feature maps of two branching networks. To verify the effectiveness of the loss function, the classification results of using only the cross-entropy loss for network training are compared with the results of using the loss function Loss (loss1+loss2). The two network models are trained with the same hyperparameter settings. The results are shown in Table 7.

As shown, the overall effectiveness of the network model training without the contrast entropy loss is inferior to that of the network model trained by the loss function with the contrast entropy loss. This indicates that the cross-entropy loss provides an optimization direction for difference feature extraction between VPI patients and non-VPI controls.

### 3.9. Comparison with the State of Art

To verify the validity of the proposed network, this subsection describes experiments with three existing classical classification networks with single-channel inputs, namely VGG16 [56], AlexNet [57], and ResNet18 [47]. The spectrograms of the oral and nasal channels are concatenated as inputs to the single-channel network. Table 8 shows the average results of the above three networks, as well as CARS-Net, on the /i/ and /u/.

The experimental results in Table 8 show that ResNet18 has higher accuracy than VGG16 and AlexNet. The CARS-Net using ResNet18 as the branching network proposed in this paper improved in accuracy, precision, recall, and F1-score compared with ResNet18 using a single channel. This indicates the effectiveness of the difference in deep features between the spectrograms of nasal- and oral-channel acoustic signals for VPI/non-VPI vowel classification at the phoneme level.

## 4. Conclusions

The rise of artificial intelligence technology has brought new solutions to the scarcity of healthcare resources. The scarcity of speech therapists has prevented large-scale assurance of diagnosis and speech disorder assessment for patients with VPI. This paper presents an automatic system for VPI detection at the subject level. Regarding VPI/non-VPI classification for unvoiced consonants, relative prominent feature description and relative feature distribution features are shown to be effective. Furthermore, the effect of unvoiced consonants with different articulatory organs on the production of VPI symptoms is explored. Regarding vowel classification, the cross-attention module embedded in CARS-Net has been proven to be effective in the VPI/non-VPI classification task. Furthermore, the sensitivity of CARS-Net for vowels with different tongue positions on VPI classification is explored. CARS-Net is more sensitive to high vowels than to vowels of lower tongue position for VPI/non-VPI classification. The experimental results obtained by voting on the phoneme level results also demonstrate the effectiveness of the proposed system for VPI recognition at the subject level. The performance of the system makes screening for VPI and assessment for speech disorders possible even in a global shortage of speech therapists.

## 5. Future Work

An automatic ancillary diagnostic approach to speech-based VPI is proposed in the work. It is the achievement of qualitative diagnosis for VPI patients. In the clinical treatment of VPI patients, the assessment of the severity of VPI is also important. This not only helps in developing treatment plans for VPI patients, but also provides an objective basis for the recovery process. In the future work, we will explore the feasibility of assessing severity of VPI patients based on speech data.

## Figures and Tables

**Table 1:** VPI detection results at the subject level (%).

Accuracy	Precision	Recall	F1-Score	TNR	FPR
98.52	97.80	100.00	98.89	95.65	4.35

**Table 2:** VPI speech classification accuracy at the phoneme level for consonants (%).

Features Set	SVM	LDA	Adaboost
RPFD	76.19	76.81	78.82
RFD	84.81	84.74	80.10
RPFD+RFD	85.00	84.85	83.30

**Table 3:** VPI speech classification accuracy at the phoneme level for different consonants (%).

Categories of Different Initial Consonants			Classifiers		
			SVM	LDA	Adaboost
bilabial	plosive	/p/	80.77	80.77	81.54
alveolar	plosive	/t/	77.69	77.69	80.00
velar	plosive	/k/	80.94	80.14	73.13
	fricative	/h/	83.33	83.33	81.05
front palatal	affricate	/q/	89.37	91.00	90.97
	fricative	/x/	91.32	92.12	88.12
blade alveolar	affricate	/c/	87.32	86.58	87.32
retroflex	fricative	/sh/	84.17	84.17	85.00
labiodental	fricative	/f/	90.11	87.83	82.56

**Table 4:** VPI/non-VPI classification results at the phoneme level for different vowels (%).

	$N_{h}=4$	$N_{h}=8$	$N_{h}=16$
Accuracy	84.81	84.32	84.80

**Table 5:** VPI/non-VPI classification result at the phoneme level for different vowels (%).

	Accuracy	Precision	Recall	F1-Score
/a/	89.73	92.06	92.92	92.19
/e/	90.36	93.92	93.55	93.42
/i/	93.72	95.42	96.11	95.45
/u/	93.42	95.40	95.25	95.10

**Table 6:** VPI/non-VPI speech classification for vowels with CA-module or not (%).

Vowel	Structure	Accuracy	Precision	Recall	F1-Score
/a/	No_CA ^1^	80.24	81.67	90.69	85.71
	CARS-Net	89.73	92.06	92.92	92.19
/e/	No_CA ^1^	88.00	90.59	92.13	91.08
	CARS-Net	90.93	93.84	93.52	93.36
/i/	No_CA ^1^	89.25	95.22	89.44	91.76
	CARS-Net	93.71	95.42	96.09	95.44
/u/	No_CA ^1^	93.13	95.09	95.22	94.89
	CARS-Net	93.42	95.39	95.27	95.11

^1^ CRAS-Net without CA module.

**Table 7:** VPI/non-VPI speech classification for vowels results with loss2 or not (%).

Vowel	Loss	Accuracy	Precision	Recall	F1-Score
/a/	loss1	71.53	86.08	74.17	76.15
	loss1+loss2	89.73	92.06	92.92	92.19
/e/	loss1	83.66	89.36	86.80	87.34
	loss1+loss2	90.93	93.84	93.52	93.36
/i/	loss1	90.79	94.16	92.43	93.20
	loss1+loss2	93.71	95.42	96.09	95.44
/u/	loss1	88.91	93.69	89.49	90.26
	loss1+loss2	93.42	95.39	95.27	95.11

**Table 8:** Comparison experiment results of VPI/non-VPI speech classification for vowels (%).

	Accuracy	Precision	Recall	F1-Score
VGG16	83.21	84.47	94.23	87.95
AlexNet	90.96	93.36	94.09	93.25
Resnet18	92.60	94.74	94.79	94.53
CARS-Net	93.57	95.41	95.67	95.27

## Data Availability

The datasets presented in this article include identifiable sound information that is privacy-preserving and must be approved by the West China Hospital of Stomatology, Sichuan University. Requests to access the datasets should be directed to L.H., ling.he@scu.edu.cn.
